# Agnuside Alleviates Synovitis and Fibrosis in Knee Osteoarthritis through the Inhibition of HIF-1*α* and NLRP3 Inflammasome

**DOI:** 10.1155/2021/5534614

**Published:** 2021-03-16

**Authors:** Li Zhang, Xiaochen Li, Haosheng Zhang, Zhengquan Huang, Nongshan Zhang, Li Zhang, Runlin Xing, Peimin Wang

**Affiliations:** ^1^The Affiliated Hospital of Nanjing University of Chinese Medicine, Department of Orthopedics, Nanjing 210029, China; ^2^Key Laboratory for Metabolic Diseases in Chinese Medicine, First College of Clinical Medicine, Nanjing University of Chinese Medicine, Nanjing 210023, China; ^3^Jiangsu Province Hospital of Chinese Medicine, Nanjing, Jiangsu 210029, China; ^4^Affiliated Hospital of Nanjing University of Chinese Medicine, Hanzhong Road 155, Nanjing, Jiangsu Province, China

## Abstract

Increasing evidence has shown that NLRP3 inflammasome activation participates in chronic aseptic inflammation and is related to tissue fibrosis. Our last study also revealed the vital role of NLRP3 inflammasome, highly associated with tissue hypoxia, in the onset and development of knee osteoarthritis (KOA). In this study, we tried to find a possible benign intervention for that pathological process. Agnuside (AGN), a nontoxic, natural small molecule isolated from the extract of *Vitex negundo L.* (Verbenaceae), has been demonstrated to have antioxidation, anti-inflammatory, analgesia, and many other properties as an iridoid glycoside, although its specific target is still unclear. Therefore, we established MIA-induced KOA model rats and investigated the effects of AGN oral gavage on oxygen-containing state, NLRP3 inflammasome, synovitis, and fibrosis in KOA. Pimonidazole staining and HIF-1*α* immunohistochemical assay both showed that AGN at the oral dose of 6.25 mg/kg can effectively relieve local hypoxia in synovial tissue. Besides, we observed a decrease of HIF-1*α*, caspase-1, ASC, and NLRP3 after AGN intervention, both in the mRNA and protein levels. In addition, rats treated with the AGN showed less inflammatory reaction and fibrosis, not only in the expression of NLRP3, inflammasome downstream factors IL-1*β* and IL-18, and fibrosis markers TGF-*β*, TIMP1, and VEGF but also in the observation of HE staining, anatomical characteristics, Sirius Red staining, and type I collagen immunohistochemistry. Subsequently, we established LPS-induced models of fibroblast-like synoviocytes (FLSs) mimicking the inflammatory environment of KOA and activating NLRP3 inflammasome. FLSs treated with AGN (3 *μ*M) resulted in a downregulation of HIF-1*α* and the components required for NLRP3 inflammasome activation. Meanwhile, the content of proinflammatory factors IL-1*β* and IL-18 in FLS supernatant was also reduced by AGN. In addition, both mRNA and protein levels of the fibrotic markers were significantly decreased after AGN management. To conclude, this study demonstrates that AGN alleviates synovitis and fibrosis in experimental KOA through the inhibition of HIF-1*α* accumulation and NLRP3 inflammasome activation. Additionally, not only does it reveal some novel targets for anti-inflammatory and antioxidant effects of AGN but also announces its potential value in treating KOA in humans.

## 1. Introduction

Chronic low-grade inflammation in synovial tissue is a major driver of the ongoing joint degeneration in knee osteoarthritis (KOA) [1]. Under this inflammatory condition, KOA synovium occurs with pathological changes such as synovitis and fibrosis, highly associated with the clinical symptoms of pain, joint swelling, and stiffness, ultimately leading to disability [2]. Moreover, synovium damage may increase oxygen consumption, setting the tissue into a state of local hypoxia, which has been extensively studied in arthritis disorders, not just KOA [3]. Subsequently, hypoxia-inducible transcription factor-1*α* (HIF-1*α*) accumulation triggers cellular responses to adapt to the anoxia environment, thus further exacerbating the development of synovitis and fibrosis [4]. In our last study, the correlation between HIF-1*α* upregulation and the nod-like receptor protein (NLRP3) inflammasome activation during KOA was investigated in vivo and in vitro. We also revealed that increased HIF-1*α* in KOA aggravates synovitis and fibrosis via NLRP3 activation [5].

The NLRP3 inflammasome is considered the most characteristic of NLR family, which contains NLR protein 3, an apoptosis-associated speck-like protein containing a caspase recruitment domain (ASC), and pro-caspase-1 [6]. The assembly and activation of the NLRP3 inflammasome will lead to an inflammatory cascade response based on the maturation and secretion of IL-1*β*, IL-18, HMGB1, and even to cell pyroptosis [7]. Two steps are generally considered to be required to complete this process, the NF-*κ*B signaling pathway activation under inflammatory stimuli and pro-caspase-1 cleaved (to cleaved caspase-1 p10 and p20) via pathogen-associated molecular patterns (PAMPs) and damage-associated molecular patterns (DAMPs). Notably, these “steps” also play a vital role in the pathology of KOA. Besides, increasing evidence has shown that NLRP3 activation in different tissues (such as the liver, renal, and airway) participates in chronic aseptic inflammation and can be related to tissue fibrosis [8–10]. Therefore, how to treat chronic inflammation via the intervention of NLRP3 inflammasome becomes a hotspot.

Synovial fibrosis, another important aspect of KOA synovial lesions, has attracted increasing attention in recent years. Its main pathological changes include extracellular matrix (ECM) accumulation and angiogenesis [11]. Numerous studies have confirmed the collagen deposition effect of transforming growth factor-*β* (TGF-*β*), while the collagen degradation inhibition is mainly due to the tissue inhibitor of metalloproteinase 1 (TIMP1) [12, 13]. They both are crucial in regulating the ECM homeostasis. Previous studies also made detailed observations of angiogenesis in KOA synovial fibrosis, which may have highly similar pannus to rheumatoid arthritis under magnetic resonance imaging, and such pathological process is highly associated with vascular endothelial growth factor (VEGF) [14, 15]. Indeed, synovial fibrosis may largely be irreversible; therefore, antifibrotic therapy aimed at slowing down the fibrosis process is of great value in clinical application in KOA.

In the long-term practice of orthopedic treatment, we found “Sanse Powder,” an external application, of traditional Chinese medicine, had a good clinical effect on treating KOA [16]. Subsequent studies explored the pharmaceutical components of “Sanse Powder” using ultraperformance liquid chromatography and identified agnuside (AGN), a nontoxic, natural small molecule isolated from the extract of *Vitex negundo L.* (Verbenaceae), as a part of medicinal effective ingredients [17]. Meanwhile, accumulating evidence has demonstrated the antioxidation, anti-inflammatory, analgesia, and many other properties of AGN as an iridoid glycoside [18–20]. Nevertheless, the medicinal effects of AGN evaluated in existing studies are still very limited, nor are the targets of AGN's efficacy clear.

In summary, KOA shows a combination of oxygen deficit and a state of low-grade inflammation, such pathological features are reflected in synovitis and fibrosis mediated by HIF-1*α* accumulation and NLRP3 activation. By targeting HIF-1*α*/NLRP3, AGN may have exerted antioxidation and anti-inflammatory effects. Accordingly, we hypothesized that AGN should alleviate synovitis and fibrosis in KOA through the inhibition of HIF-1*α* and NLRP3 inflammasomes.

## 2. Materials and Methods

### 2.1. In Vivo Animal Experimental Design

Twenty-four 2-month-old SD male rats, weight ranging from 210 g to 250 g (provided by Beijing Vital River Laboratory Animal Technology Co., Ltd.), were used. Animals were housed in a specific pathogen-free, laminar-flow housing apparatus under controlled temperature, humidity, and 12 h light/dark regimen and maintained on a standard rodent pellet diet. All animal protocols were approved by the Animal Care and Use Committee of the Nanjing University of Chinese Medicine. All experiments were conducted in accordance with the National Institutes of Health Guidelines for the Care and Use of Laboratory Animals. After one week of adaptive feeding, the first day of the experiment was recorded as Day 1.

Rats were randomly assigned to three groups: normal, KOA, and KOA+AGN, with eight rats in each group. On Day 1, the KOA model was constructed by inducing by intra-articular injection of 1 mg monosodium iodoacetate (MIA) as described previously, on both knees. 14 days after surgery (Day 14), the KOA model was successfully established, and drug administration began. 6.25 mg of AGN (Yuanye Bio-Technology Co., Ltd., Shanghai, China; HPLC > 98%) was prepared as suspension in 0.5% *w/v* sodium carboxymethyl cellulose in 10 ml sterilized physiologic saline. The KOA+AGN group received an oral dose (1 ml/100 g body weight/1 day) by oral gavage, while the other two groups received an equal volume of sodium carboxymethyl cellulose solution. After 21 days of AGN administration (Day 35), all rats were anesthetized with Nembutal, then abdominal aortic serum and synovial tissues were collected. The dosage, concentration, and intervention time of AGN are all referred to the previous study [19, 20].

### 2.2. Histological Analysis

Synovial tissues were fixed in 10% neutral formalin after rats were executed, embedded in paraffin, and cut into slices for routine staining. We followed Kenn's criteria in scoring and analyzing sections to evaluate synovitis [21].

Sirius Red staining was carried out according to the instructions of Sirius Red Stain kit (Beyotime Biotechnology, Shanghai, China). Sections were mounted and viewed under a Leica DMI3000B microscope (Leica, Germany), with the use of bright fields.

### 2.3. Immunohistochemical Assay

Immunohistochemistry stainings were performed to observe the expression of HIF-1*α* and type I collagen in synovial tissue. We followed the methods of Peimin Wang et al. [22]. Briefly, sections were incubated in antigen retrieval buffer to unmask the antigen after a standard deparaffinization and rehydration process. Sections were treated with 3% hydrogen peroxide for 10 min. After that, the sections were permeabilized with 0.1% Triton X-100 in PBS for 5 min at room temperature, blocked with 1% BSA at room temperature for 1 h, and then incubated with primary antibodies (in 1% BSA, 0.1% Triton X-100) at 4°C overnight. For secondary reactions, species-matched HRP-labelled secondary antibody was used (1 : 500 in 1% BSA, 1 h) at 37°C. DAB was used as chromogen, and hematoxylin was used to counterstain. High-quality images were captured in six fields per sample, and semiquantitative analysis was measured by determining percentage of positive areas with ImageJ.

### 2.4. Pimonidazole Staining and Immunofluorescence

To investigate synovium tissue hypoxia, rats were injected with pimonidazole HCl (Hypoxyprobe™-1 Plus Kit, Burlington, MA, USA) at a dosage of 60 mg/kg for 45 min prior to sacrifice as previously described [4]. Subsequent immunofluorescence staining followed the kit instructions. The image was observed by inverted fluorescence microscope (Leica DMI3000B, Germany).

### 2.5. Cell Preparation and Treatment

Primary rat fibroblast-like synoviocytes (FLSs) were obtained from additional normal rats. In brief, synovial tissues were washed for 2-3 times with phosphate-buffered saline (PBS) and then minced into pieces of 2-3 mm^2^, digested in 0.1% collagenase type II (Sigma, St. Louis, MO, USA) for 30 min. Following cell dissociation, the samples were filtered through a cell strainer. After dissociation, fibroblasts were pelleted by centrifugation at 1500 rpm for 4 min and cultured in DMEM supplemented with 10% fetal bovine serum (FBS; Gibco, Thermo Fisher Scientific, Waltham, MA, USA) and antibiotics (100 U/ml penicillin, 100 *μ*g/ml streptomycin; Invitrogen, CA, USA). Cells were identified as our previous study [23]. Passages 3-6 of the synovial fibroblasts were used for the experiments.

Lipopolysaccharide (LPS), obtained from Sigma-Aldrich (St Louis, MO, USA), was used to simulate the inflammatory environment of KOA and activate the NLRP3 inflammasome. FLSs were stimulated with LPS (10 *μ*g/ml) in DMEM for 6 h as the KOA group or exposed to DMEM with the same volume of PBS served as the normal group. The LPS+AGN group was treated with AGN (3 *μ*M) for 24 h after the challenge of LPS, while the remaining groups were treated with PBS. The concentration and intervention time of AGN are all referred to the previous study [19, 20].

### 2.6. *Caspase-1* Activity Analysis

After the treatment of AGN and/or LPS, FLSs were harvested and used to detect caspase-1 activity via Caspase-1 Activity Assay Kit (Beyotime Biotechnology, Shanghai, China) according to the manufacturer's instructions. The absorbance of the samples was measured at 405 nm using a microplate reader (PerkinElmer EnSpire, USA). All samples were quantified following comparison to the normal group and calculated the relative changes of caspase-1 activity.

### 2.7. ELISA Assay

IL-1*β* and IL-18 levels in the rat serum and culture media were determined using a commercially available rat IL-1*β* and IL-18 enzyme-linked immunosorbent assay (ELISA) kit (Nanjing Jin Yibai Biological Technology Co. Ltd., Nanjing, China) according to the manufacturer's instructions.

### 2.8. Western Blotting

Briefly, synovial tissues or FLSs were mixed with RIPA lysate and grinded for 10-15 min, respectively. The protein levels were quantified with a BCA protein assay kit (Beyotime Biotechnology, Shanghai, China). Then, the samples were electrophoresed in SD-PAGE to separate protein bands. Proteins were transferred from the gel onto PVDF membranes and blocked with 5% nonfat dry milk for 2 h. The membrane was incubated with the first antibody (1 : 1000, Abcam, Cambridge, UK) for overnight at 4°C and then a second antibody (Thermo Fisher Scientific, Shanghai, China) for 2 h.

Later, bands were visualized by exposure to ECL method, and the overall gray value of protein bands was quantified actin as an internal marker, namely, target protein gray value/internal reference overall gray value.

### 2.9. Quantitative Real-Time PCR

We followed the methods of Wang et al. [24, 25]. Total RNA was extracted with TRIzol and assessed by spectrophotometer. Then, reverse transcription of RNA from each group was performed using Prime Script RT reagent Kit (Beyotime Biotechnology, Shanghai, China). Primer was designed and synthesized by Shanghai Biotechnology Service Company in accordance with Gene sequence in GenBank Gene sequence design, together with Oligo v6.6 (sequences as Table 1). qPCR was performed using Premix Ex Taq SYBR-Green PCR (Takara) according to the manufacturer's instructions on an ABI PRISM 7300 (Applied Biosystems, Foster City, CA, USA). The mRNA level of individual genes was normalized to GAPDH and calculated by 2^−*ΔΔ*CT^data analysis method.

### 2.10. Statistical Analysis

Statistical analysis was performed using GraphPad Prism 6.0 Software (San Diego, CA, USA). Data are presented as mean ± standard deviation (SD). Group comparisons were assessed with one-way ANOVA or Student's *t*-test with Bonferroni's post hoc test for comparison of multiple columns. A value of *P* < 0.05 (two-tailed) was considered statistically significant. Higher significance levels were established at *P* < 0.01.

## 3. Results

### 3.1. Agnuside Relieves the State of Hypoxia in KOA Rats

**Figure 1:**
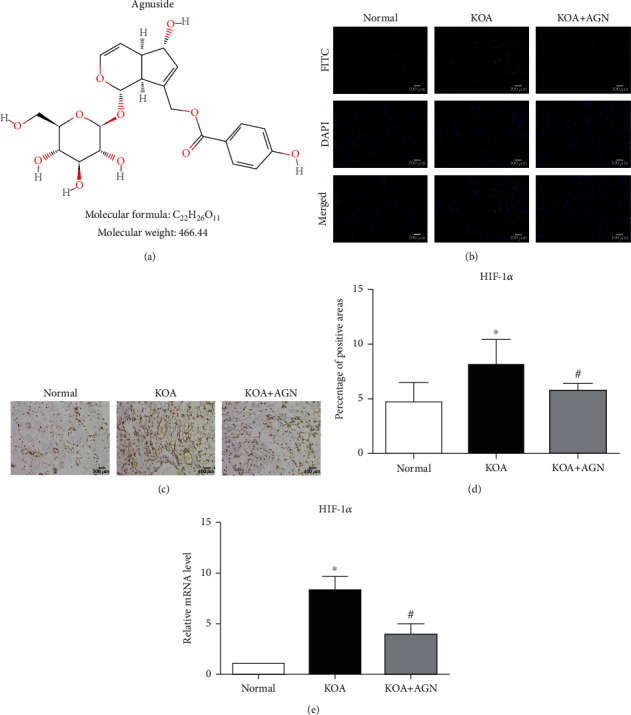
Agnuside relieves the state of hypoxia in KOA rats. (a) The chemical structure of AGN. (b) Representative synovial tissues stained with pimonidazole, 200x, scale bar = 100 *μ*m. (c) Representative HIF-1*α* immunohistochemical sections of synovial tissues in each group, 200x, scale bar = 100 *μ*m. (d) The percentage of HIF-1*α*-positive areas in each group. Data were analyzed by ImageJ. ^∗^*P* < 0.05, compared with the normal group. ^#^*P* < 0.05, compared with the KOA group. (e) mRNA level of HIF-1*α* between groups. ^∗^*P* < 0.05 compared with the normal group. ^#^*P* < 0.05, compared with the KOA group.

The chemical structure of AGN is shown as Figure 1(a). Similar to our previous study, the synovial tissues of MIA-induced KOA model rats showed aggravated hypoxia compared with the normal group observed by pimonidazole staining. AGN (6.25 mg/kg) could be able to relieve this situation (Figure 1(b)). Subsequently, immunohistochemistry was performed to evaluate the protein expression of HIF-1*α* in synovial tissue (Figures 1(c) and 1(d)). The percentage of HIF-1*α*-positive areas in the KOA group showed a significant upregulation compared with the normal group (*P* < 0.05), while the KOA+AGN group showed a significant downregulation compared with the KOA group (*P* < 0.05). The mRNA levels of HIF-1*α* measured by PCR in each group were consistent with the immunohistochemistry assay (Figure 1(e)).

### 3.2. Agnuside May Inhibit NLRP3 Inflammasome and Alleviate Synovitis in KOA Rats

**Figure 2:**
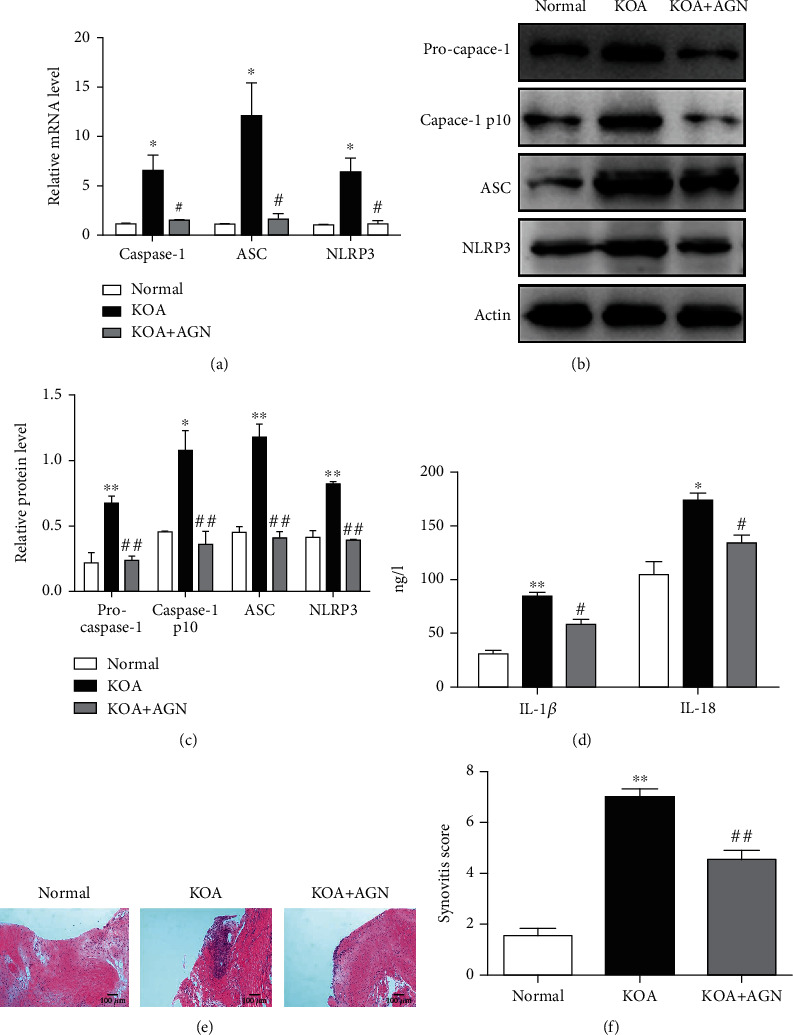
Agnuside may inhibit NLRP3 inflammasome and alleviate synovitis in KOA Rats. (a) mRNA levels of caspase-1, ASC, and NLRP3 in each group. ^∗^*P* < 0.05, in comparison with the normal group. ^#^*P* < 0.05, in comparison with the KOA group. (b) Typical protein bands for each group. (c) Protein level comparison of pro-caspase-1, caspase-1, p10, ASC, and NLRP3 between groups. ^∗^*P* < 0.05, ^∗∗^*P* < 0.01, compared with the normal group. ^##^*P* < 0.01, compared with the KOA group. (d) Serum levels of IL-1*β* and IL-18 between groups. ^∗^*P* < 0.05, ^∗∗^*P* < 0.01, compared with the normal group. ^#^*P* < 0.05, compared with the KOA group. (e) Representative synovial tissues of each group stained with stained with HE staining, 200x, scale bar = 100 *μ*m. (f) Synovitis score according to Kenn's criteria. ^∗∗^*P* < 0.01, compared with the normal group. ^##^*P* < 0.01, compared with the KOA group.

To observe the effect of AGN on NLRP3 inflammasome in KOA, PCR and WB were performed to quantitatively study the expression of pro-caspase-1, caspase-1 p10, ASC, and NLRP3 protein (Figures 2(a)–2(c)). Both the mRNA and protein levels of these substances in the KOA group were significantly higher than the normal group (*P* < 0.05), and the KOA+AGN group resulted in a reduced expression compared with the KOA group (*P* < 0.05). Subsequently, we analyzed the serum content of IL-1*β* and IL-18 in each group with ELISA (Figure 2(d)). As the downstream of NLRP3 inflammasome activation, these proinflammatory factors resulted in an upregulation in the KOA group compared with the normal group (*P* < 0.05) while the KOA+AGN group showed a significant downregulation compared with the KOA group (*P* < 0.05). Besides, we evaluated synovial inflammation overall in all groups of rats. Under the observation of HE sections, the KOA+AGN group showed orderly arranged synovial lining cells, loose connective tissue, and less inflammatory cell infiltration compared with the KOA group (Figure 2(e)). Same results were obtained from the synovitis score according to Kenn's criteria in scoring HE sections (Figure 2(f)).

### 3.3. Agnuside Alleviates Synovial Fibrosis in KOA Rats

**Figure 3:**
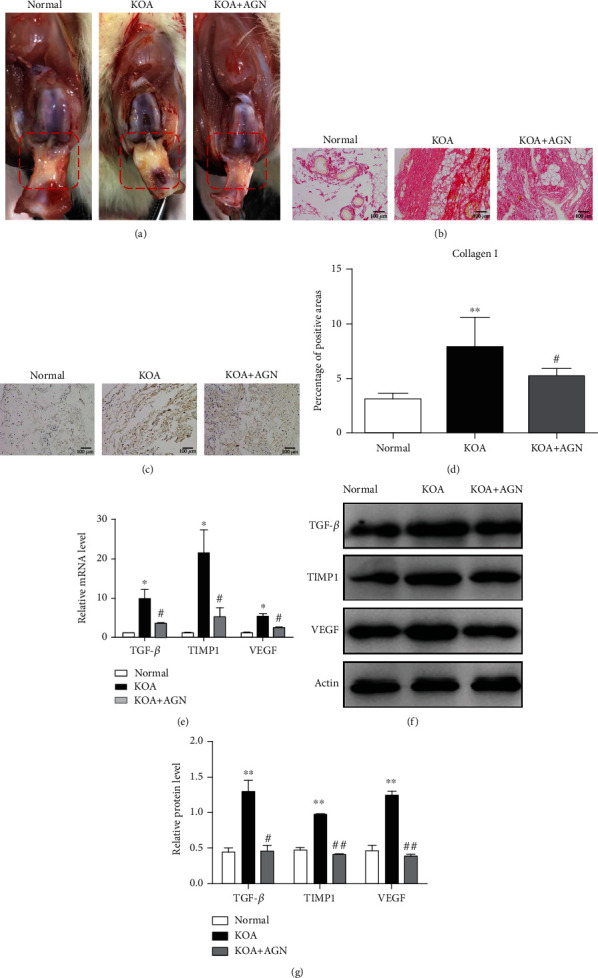
Agnuside alleviates synovial fibrosis in KOA rats. (a) Anatomical changes of synovial tissues (red box area) in each group. (b) Collagen deposition was revealed through Sirius Red staining, 200x, scale bar = 100 *μ*m. (c) Representative type I collagen immunohistochemical sections of synovial tissues in each group, 200x, scale bar = 100 *μ*m. (d) Semiquantification of immunohistochemical sections was evaluated by calculating the percentage of collagen I-positive areas. ^∗∗^*P* < 0.01, in comparison with the normal group. ^#^*P* < 0.05, in comparison with the KOA group. Data were analyzed by ImageJ. (e) Comparison of TGF-*β*, TIMP1, and VEGF mRNA levels between groups. ^∗^*P* < 0.05, compared with the normal group. ^#^*P* < 0.05, compared with the KOA group. (f) Typical protein bands for each group. (g) Comparison of TGF-*β*, TIMP1, and VEGF protein expressions between groups. ^∗∗^*P* < 0.01, compared with the normal group. ^#^*P* < 0.05, ^##^*P* < 0.01, compared with the KOA group.

To evaluate the effect of AGN on synovial fibrosis in KOA, anatomical characteristics and pathological sections of synovial tissue were observed. The KOA group showed markedly increased collagen deposition, while this change was relatively lessened in the KOA+AGN group observed under anatomy (Figure 3(a)). The same results can be observed by Sirius Red staining (Figure 3(b)). Subsequently, we performed a semiquantitative analysis by immunohistochemical staining of type I collagen (Figures 3(c) and 3(d)). In the KOA group, the percentage of collagen I-positive areas was significantly higher in comparison with the normal group (*P* < 0.05). But in the KOA+AGN group, collagen I deposition was significantly alleviated compared with the KOA group (*P* < 0.05). More specifically, we measured both mRNA and protein expressions of fibrotic markers TGF-*β*, TIMP1, and VEGF in synovial tissues (Figures 3(e)–3(g)). We found that there was a significant decrease level of profibrotic substances in the KOA+AGN group.

### 3.4. Agnuside Inhibits HIF-1*α* Accumulation and NLRP3 Inflammasome Activation in LPS-Treated FLSs

**Figure 4:**
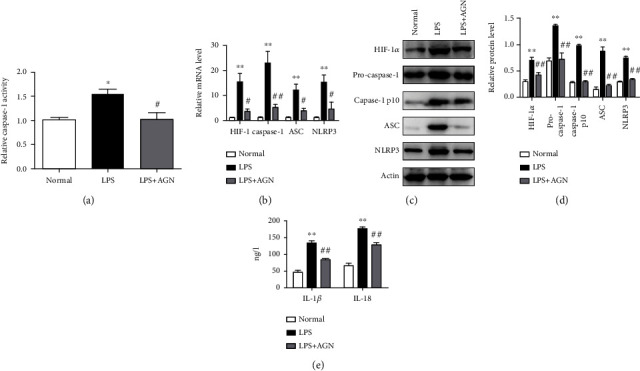
Agnuside inhibits HIF-1*α* accumulation and NLRP3 inflammasome activation in LPS-treated FLSs. (a) Relative caspase-1 activity for each group. ^∗^*P* < 0.05, in comparison with the normal group. ^#^*P* < 0.05, in comparison with the LPS group. (b) mRNA level of HIF-1*α*, caspase-1, ASC, and NLRP3 in each group. ^∗^*P* < 0.05, ^∗∗^*P* < 0.01, in comparison with the normal group. ^#^*P* < 0.05, ^##^*P* < 0.01, in comparison with the KOA group. (c) Typical protein bands for each group. (d) Protein level comparison of HIF-1*α*, pro-caspase-1, caspase-1 p10, ASC, and NLRP3 between groups. ^∗∗^*P* < 0.01, compared with the normal group. ^##^*P* < 0.01, compared with the KOA group. (e) FLS supernatant contents of IL-1*β* and IL-18 between groups. ^∗∗^*P* < 0.01, compared with the normal group. ^##^*P* < 0.01, compared with the KOA group.

The LPS-induced model of FLSs was established mimicking the inflammatory environment of KOA and activating NLRP3 inflammasome. We first observed the effect of AGN on the caspase-1 activity in KOA FLSs (Figure 4(a)). LPS stimulation significantly increased the activity of caspase-1 in normal FLSs, and AGN (3 *μ*m) significantly reduced the increased caspase-1 activity of LPS challenge (*P* < 0.05). Furthermore, we investigated the effects of AGN on HIF-1*α* and NLRP3 inflammasome components in vitro (Figures 4(b)–4(d)). In the LPS group, both mRNA and protein levels of HIF-1*α* were upregulated compared with the normal (*P* < 0.05), AGN significantly prevented this upregulation (*P* < 0.05). LPS also promoted the mRNA expression of caspase-1, ASC, and NLRP3, and this trend was reversed by AGN. Same changes occurred in protein expression, not only the precursors of caspase-1 but also the cleavage caspase-1 p10. In addition, we studied the effect of AGN on the downstream product of NLRP3 inflammasome activation. The content of IL-1*β* and IL-18 (Figure 4(e)) in the supernatant of the LPS+AGN group was significantly lower than that in the LPS group (*P* < 0.05).

### 3.5. Agnuside Downregulates Fibrosis Marker Expression in LPS-Treated FLSs

**Figure 5:**
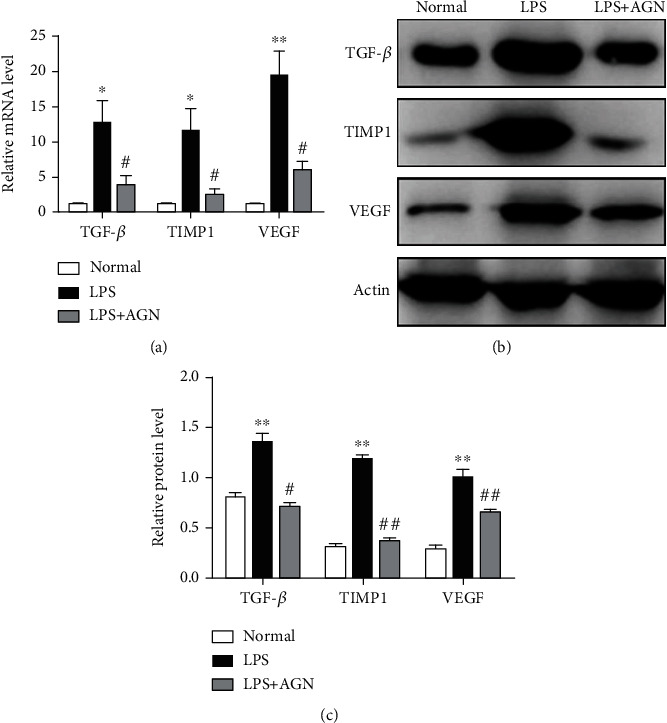
Agnuside downregulates fibrosis marker expression in LPS-treated FLSs. (a) Relative mRNA levels of TGF-*β*, TIMP1, and VEGF in FLSs in each group. ^∗^*P* < 0.05, ^∗∗^*P* < 0.01, in comparison with the normal group. ^#^*P* < 0.05, in comparison with the KOA group. (b) Typical protein bands for each group. (c) Comparison of TGF-*β*, TIMP1, and VEGF protein expressions between groups. ^∗∗^*P* < 0.01, compared with the normal group. ^#^*P* < 0.05, ^##^*P* < 0.01, compared with the KOA group.

After AGN-treated for 24 hours, both mRNA and protein expressions of TGF-*β*, TIMP1, and VEGF in FLSs were assessed (Figures 5(a)–5(c)). These fibrosis markers showed a significant upregulation in the LPS group compared with the normal group (*P* < 0.05), and the LPS+AGN group resulted in a downregulation compared with the LPS group (*P* < 0.05).

## 4. Discussion

The current study indicates that AGN may alleviate synovitis and fibrosis in KOA through the inhibition of HIF-1*α* accumulation and NLRP3 inflammasome activation. According to our literature search, the number of studies on AGN is very small. Fortunately, these few studies have provided us with the effective dose of AGN in vivo and in vitro, the time of intervention, distribution in vivo, and so on, which provide great convenience for our work [19, 20]. Therefore, we chose an oral dose at 6.25 mg/kg and a cellular dose of 3 *μ*M to continue our experiment. We select MIA intra-articular injection to construct the KOA model. MIA induces cartilage degeneration and subchondral bone sclerosis, which mimics the pathological changes observed in human OA [26]. More importantly, synovitis and fibrosis in the MIA method develop rapidly compared with surgical modeling such as anterior cruciate ligament or meniscus resection; it could be more suitable to research the effect of AGN intervention. For the same reason, we selected LPS at the dose of 10 *μ*g/ml to operate in vitro experiments.

To the best of our knowledge, this may be the first study to reveal the target of AGN efficacy. Previous studies have focused more on the extraction and purification of AGN from a chaste trees (Vitex agnus cactus L., Family Verbenaceae), or a preliminary observation on the anti-inflammatory and antioxidant capacity of AGN. In this study, we revealed that HIF-1*α* and NLRP3 inflammasomes are effective intervention targets for AGN. We also conducted a series of quantitative studies to demonstrate that AGN prevents HIF-1*α* accumulation and NLRP3 inflammasome activation, thereby alleviating KOA synovitis and fibrosis. Since the symptoms of KOA are directly related to the severity of synovitis and fibrosis, the findings of this paper may suggest a potential value in KOA treatment.

As a motor joint, it is widely accepted that the local tissue of the KOA joint is deficient in oxygen [27]. When oxygen demand exceeds supply, a cascade of intracellular events is activated, increasing the expression of HIF-1*α*. Qing et al. proved that the expression of HIF-1*α* in the synovial fluid and articular cartilage is associated with the disease severity in KOA [28]. HIF-1*α* promotes the development of inflammation; besides, as a transcription factor, HIF-1*α* directly regulates the expression of genes encoding proteins involved in fibrosis, such as TGF-*β* and VEGF [29]. Thus, hypoxia may be common upstream of synovitis and fibrosis in KOA. A variety of plant extracts have antioxidant properties, in our study; AGN, a compound isolated from the extract of Vitex negundo L., significantly improved the oxygen-containing state of synovial tissue in KOA rats. Our findings are based on the direct observation of anoxic probes and the quantitative detection of HIF-1*α*; in vitro experiments with FLSs yielded the same conclusions. These evidences suggest that AGN can reduce the accumulation of HIF-1*α*.

NLRP3 inflammasome activation may be one of the consequences of HIF-1*α* accumulation [30]. How to relieve local inflammation by inhibiting NLRP3 has also become a focus of anti-inflammatory therapy due to the critical role of NLRP3 in mediating inflammatory cascade amplification. A variety of herbal or herbal extracts have been confirmed benign intervention on NLRP3 inflammasome. Wang et al. studied the effect of isochlorogenic acid A intervention on NLRP3 inflammasome in an acute lung injury model and proved the expressions of NF-*κ*B/NLRP3 signaling pathway were inhibited by isochlorogenic acid A [31]. Huang et al. demonstrated that salvianolic acid B suppressed oxidative stress and neuroinflammation via regulating NLRP3 inflammasome activation [32]. Besides, Coptidis Rhizoma and others all have anti-inflammatory effects by targeting NLRP3 inflammasome [33–35]. In this study, we confirmed that AGN decreased caspase-1 activity and the protein level of cleaved caspase-1 (caspase-1 p10). Moreover, AGN was able to reduce both gene and protein expressions of caspase-1, ASC, and NLRP3 proteins, which assemble NLRP3 inflammasome during activation. Meanwhile, proinflammatory factors IL-1*β* and IL-18, downstream of NLRP3 inflammasome activation, were also downregulated after AGN intervention. Our results indicate that AGN may attenuate synovitis by inhibiting NLRP3 activation in KOA.

Increasing evidence has shown that NLRP3 inflammasome activation in different tissues such as the liver, renal, and airway participates in chronic aseptic inflammation and can be related to tissue fibrosis. The response to TGF-*β* is a key event in the onset of synovial fibrosis in KOA, in addition to TIMP1 and VEGF, which are important indicators for evaluating synovial fibrosis [36]. Antifibrosis therapy can play a positive role in the prevention and treatment of joint stiffness. Accordingly, we first examined the effect of AGN on fibrosis under anatomical observations; synovial tissue in rats with AGN intervention showed less collagen deposition. The same results can be observed by Sirius Red staining or immunohistochemical staining of type I collagen. Subsequently, we measured both the mRNA and protein expressions of TGF-*β*, TIMP1, and VEGF to evaluate fibrosis in vivo and in vitro. AGN-treated group showed a downregulation of all these profibrotic substances compared to the KOA group. Our results support the antifibrotic effect of AGN, which is likely to be achieved by inhibiting NLRP3 inflammasome activation.

The current study still has a few limitations. First, the effects of different doses or concentrations of AGN on KOA synovitis and fibrosis were not examined. This is because on the one hand, other studies have made a detailed observation; on the other hand, we were not very sure whether AGN can act on HIF-1*α* or NLRP3 inflammasome. Secondly, we did not set up a positive control group, and the difference in efficacy between monomers was not the focus of our study. Besides, the sample size of experimental animals would be bigger if possible.

## 5. Conclusion

In summary, this study demonstrates that AGN alleviates synovitis and fibrosis in experimental KOA through the inhibition of HIF-1*α* accumulation and NLRP3 inflammasome activation. Additionally, not only does it reveal some novel targets for anti-inflammatory and antioxidant effects of AGN but also announces its potential value in treating KOA in humans.

## Figures and Tables

**Table 1:** Nucleotide sequences of primers used for RT-PCR amplification.

Target gene	Forward primer	Reverse primer
HIF-1*α*	CCGCAACTGCCACCACTGATG	TGAGGCTGTCCGACTGTGAGTAC
Caspase-1	ATGGCCGACAAGGTCCTGAGG	GTGACATGATCGCACAGGTCTCG
NLRP3	GAGCTGGACCTCAGTGACAATGC	ACCAATGCGAGATCCTGACAACAC
ASC	AGAGTCTGGAGCTGTGGCTACTG	ATGAGTGCTTGCCTGTGTTGGTC
TGF-*β*	GCAACAATTCCTGGCGTTACCTTG	TGTATTCCGTCTCCTTGGTTCAGC
VEGF	AGCGTTCACTGTGAGCCTTGTTC	CCGCCTTGGCTTGTCACATCTG
TIMP1	GCGTTCTGCAACTCGGACCTG	GTGTAGGCGAACCGGATATCTGTG
GAPDH	GGCCTTCCGTGTTCCTACC	ACTCGACACCTGCCCTCA

## Data Availability

The data used to support the findings of this study are available from the corresponding author upon request.

## Abbreviations

KOA: Knee osteoarthritis
HIF-1*α*: Hypoxia-inducible transcription factor-1*α*
NLRP: Nucleotide-binding and oligomerization domain-like receptor family pyrin domain-containing
ASC: Apoptosis-associated speck-like protein with a caspase-recruitment domain
AGN: Agnuside
TGF-*β*: Transforming growth factor-*β*
TIMP1: Tissue inhibitor of metalloproteinases 1
VEGF: Vascular endothelial growth factor
FLSs: Fibroblast-like synoviocytes
LPS: Lipopolysaccharide
ATP: Adenosine triphosphate.
